# Supplementary figures and images for: Biological control of important fungal diseases of potato and raspberry by two Bacillus velezensis strains
Source: PeerJ. 2021 Jun 14;9:e11578. doi: 10.7717/peerj.11578 (PMC8210809; doi:10.7717/peerj.11578)

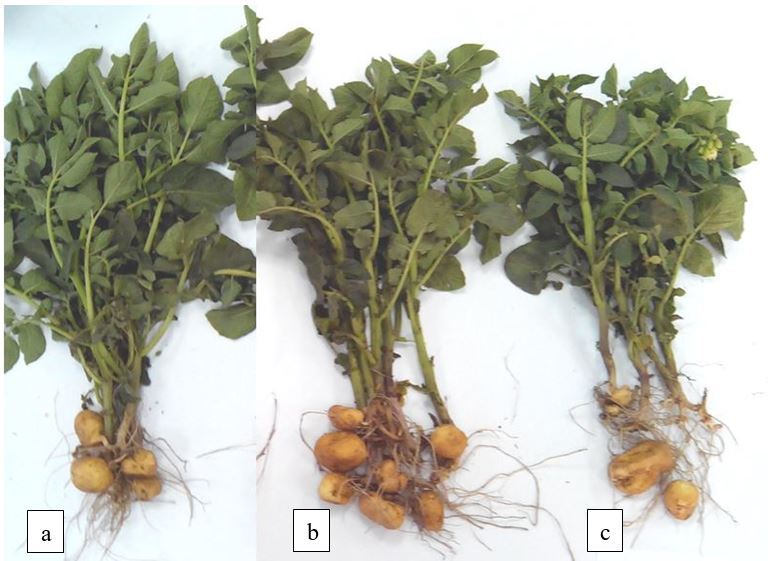


Figure S1. Potato plants cv. Yuna, 6 weeks after planting:

a – control; b – BZR 336g; c – BZR 517

Supplement: Supplemental Information 2 — A,B-antifungal activityof B. velezensis against R. solani in vitro; C, D-antifungal activity of B. velezensis against D. applanata in vitro a-104 CFU/mL, b-105 CFU/mL, c-106 CFU/mL, d–control [file peerj-09-11578-s002.docx]

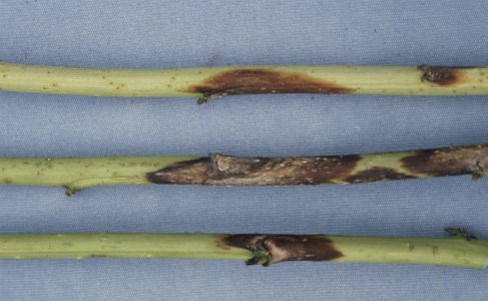


Figure S2. Raspberry canes damaged by *D. applanata*

Supplement: Supplemental Information 3 [file peerj-09-11578-s003.docx]
